# Development of a Device for Monitoring Erosion in the Field

**DOI:** 10.3390/mi15070880

**Published:** 2024-07-04

**Authors:** Thiago Augusto Mendes, Juan Félix Rodriguez Rebolledo, Sávio Aparecido dos Santos Pereira, Marcus Vinicius Miguel de Oliveira, Klebber Teodomiro Martins Formiga

**Affiliations:** 1 Graduate Program in Technology, Management and Sustainability (PPGTGS), Department of Academic Areas III, Instituto Federal de Educação, Ciência e Tecnologia de Goiás (IFG), Goiânia 74968-755, GO, Brazil; 2 Technology College, University of Brasília, Brasília 70910-900, Brazil; jrodriguezr72@hotmail.com; 3 School of Civil and Environmental Engineering, Universidade Federal de Goiás (UFG), Goiânia 74605-220, GO, Brazil; savioaparecido1@gmail.com (S.A.d.S.P.); klebberformiga@ufg.br (K.T.M.F.); 4 Graduate Program in Production and Systems Engineering (MEPROS), Pontifícia Universidade Católica de Goiás (PUC Goiás), Goiânia 74605-220, GO, Brazil; marcusvinoli@gmail.com

**Keywords:** geotechnical monitoring, soil erosion, erodibility, instrumentation, rainfall sensors

## Abstract

Monitoring erosion is an important part of understanding the causes of this geotechnical and geological phenomenon. In order to monitor them, it is necessary to develop equipment that is sophisticated enough to resist the sun and water without damage, that is self-mechanized, and that can support the amount of data collected. This article introduces a rain-triggered field erosion monitoring device composed of three main modules: control, capture, and sensing. The control module comprises both hardware and firmware with embedded software. The capture module integrates a camera for recording, while the sensing module includes rain sensors. By filming experimental soil samples under simulated rain events, the device demonstrated satisfactory performance in terms of activation and deactivation programming times, daytime image quality without artificial lighting, and equipment protection. The great differences about this monitoring device are its ease of use, low cost, and the quality it offers. These results suggest its potential effectiveness in capturing the progression of field erosive processes.

## 1. Introduction

Erosion is a geological phenomenon directly related to local geological changes. This phenomenon has been increasing due to anthropogenic interference and can cause various damages, such as siltation and sediment deposition in reservoirs associated with the erosive process at their edges. The erosion process is related to the ability of water, wind, or waves to detach and transport soil particles [1]. Several factors can interfere with this process, such as an imbalance in soil–atmosphere dynamics, fluctuations in water levels in reservoirs due to water demand and seasonality, and human activities such as boat movements, unregulated occupation of slopes, and the launching of inadequate storm drains [2,3].

Given the increase in erosive processes due to anthropogenic activities, it is important to monitor and study erosion through laboratory and field tests. Laboratory and field tests to evaluate soil erodibility include the Inderbitzen test [4,5,6], rainfall simulators [7,8,9,10], hydraulic channels, and disaggregation [11,12]. Although these tests are widely used in the geotechnical field to study soil erodibility, there is a great variety in the equipment used, with different characteristics of the specimens and the hydraulic conditions adopted [13].

Understanding and diagnosing the formation and evolution of erosion processes in the field is of great importance, especially for understanding the physical behavior of the phenomenon and supporting numerical modeling. For example, Zhu [14] used precipitation monitoring, erosion, and soil characterization data from the Dankiangkou reservoir in a GIS environment in conjunction with the Universal Soil Loss by Erosion (USLE) equation to assess the risk of erosion at the reservoir edge.

In order to obtain adequate information on the erosion process, it is necessary to advance in the development of low-cost embedded equipment and software to monitor the initial, evolutionary, and stabilization processes, if any, of erosion in the field. This equipment can support and complement the results of laboratory (experimental) tests and provide reliable geotechnical parameters for numerical modeling of erosion processes.

Some examples in the literature show the importance of developing this type of equipment for monitoring erosion processes [15,16,17,18]. Guo et al. [15] presented a suite of technologies employed for the Three Gorges Reservoir, China, to monitor soil erosion and nonpoint source pollution, including sensors with real-time transmission and predictive algorithms. Knox and Mittelstet [17] presented a non-invasive ultrasonic sensor with a high temporal resolution for monitoring soil erosion by the distance traveled by the signal pulse emitted by the sensor, with an observed accuracy of ±6.12 to 52.42 mm for field measurements. Sepuru and Dube [19] presented a literature review on the application of satellite remote sensors for mapping and monitoring erosion processes, highlighting that erosion monitoring is largely focused on the level of municipalities or river basins and that more detail is needed to assess the spatial variation and extent of erosion within a given region.

Based on the above, the main objective of this article is to present the development of a device designed to monitor the erosion process in the field by detecting rainfall events. The goal is to create an automated, low-cost, high-quality device for practical erosion monitoring. This device includes camera control hardware for automatic video image capture and custom software to manage the hardware system. It will be tested in real-world erosion scenarios to evaluate its effectiveness.

## 2. Materials and Methods

**Figure 1:**
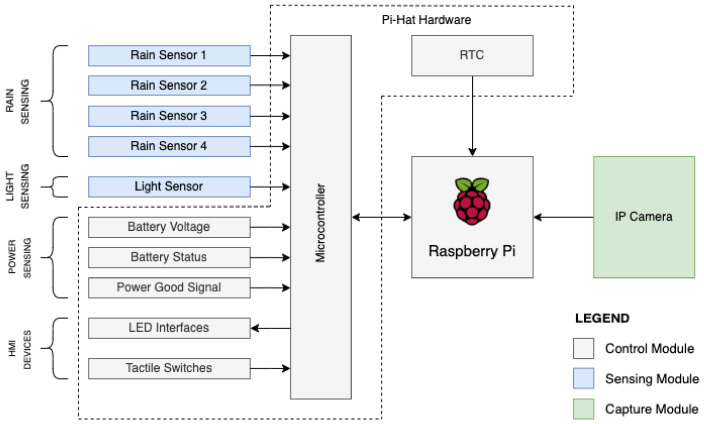
Modules for field monitoring of erosion processes developed to make up the device.

The device for monitoring erosion processes in the field has been designed considering the creation of three parts (Figure 1): the sensing module, the capture module, and the control module. The sensing module is responsible for detecting rain. It is composed of an arrangement of plates whose ohmic resistance varies according to the number of water drops (with dissolved ions) that are poured over them. Up to four rain sensors can be connected to the device to ensure that the rain phenomenon is occurring. The capture module is responsible for capturing the event of the erosive phenomenon after automatic triggering and consists of a digital surveillance video camera used for the collection of images. The control module is responsible for coordinating all device operations. It polls data from the sensing module, applies pre-defined rules to this piece of information to confirm a rain event, and then records data from the capture module. It also provides a Human Machine Interface (HMI) to configure its operations and obtain recorded video data. 

The way the three modules work together is simple: rain sensors (the sensing module) are distributed over the area of erosion to be assessed, and a camera (the capture module) is installed to record the behavior of the erosion process when rain events occur. When a rain event occurs, the raindrops fall on the rain sensors, and the system (the control module) activates the recording module, which starts recording the video for the entire period of the rain event. It is also possible to configure a delay to record images even after the rain stops.

### 2.1. Control Module

The control module consists of hardware and firmware that interfaces a single-board computer (SBC) to the rest of the system. The hardware and firmware components are described and discussed in the following sections.

#### 2.1.1. Hardware

**Figure 2:**
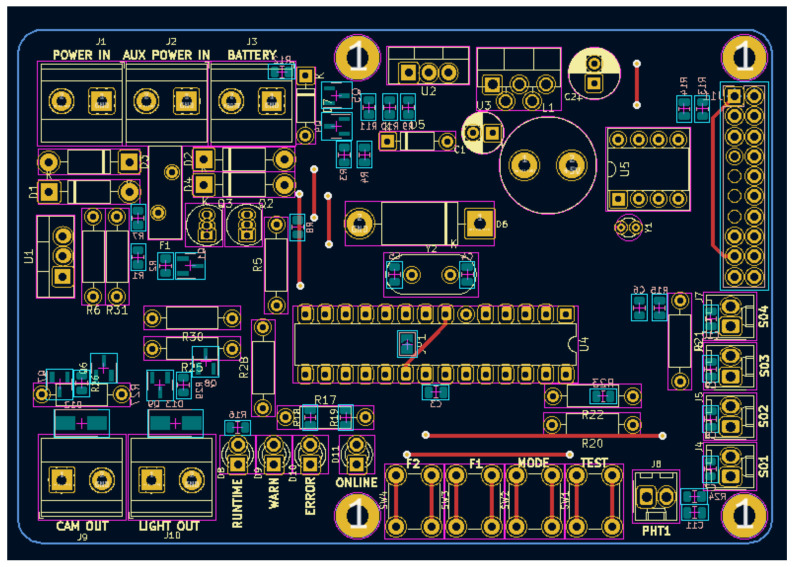
A 2D model of the Pi Hat circuit in the Kicad program, version 5.1.6.

To build the hardware, we designed a Pi Hat, a printed circuit board that sits on top of the Raspberry Pi (the chosen SBC for this paper) like a hat. This board has many submodules: A simple uninterruptible power supply (UPS) to provide electrical power to both the Raspberry and the camera from an external rechargeable battery or power adaptor; a real-time clock (RTC) to provide reliable information of date and time; LEDs for status information; tactile buttons for further implementations; and mainly, a microcontroller (MCU) that reads analog data from sensors and delivers it to the Raspberry Pi over the UART interface. The 2D model of the circuit is shown in Figure 2 (the bottom cooper traces are omitted).

**Figure 3:**
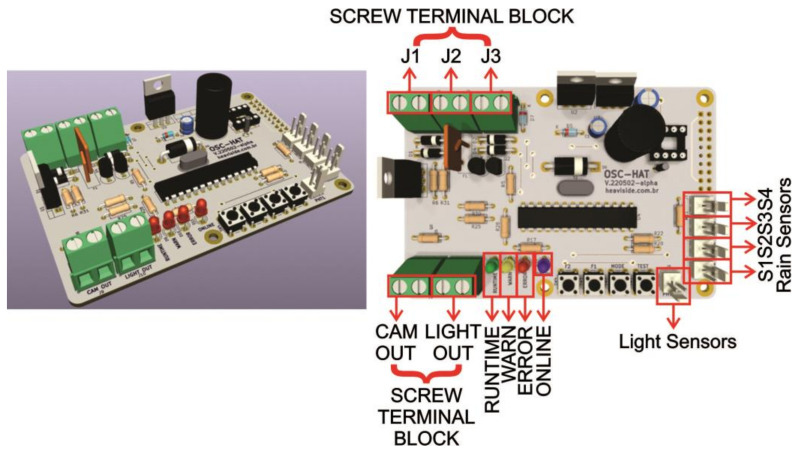
Electronic project requirements: interface board with description of its necessary elements.

The interface board is shown in Figure 3. At the bottom, LEDs indicate the operating status, and each status is detailed in Table 1.

The rain sensors are connected via connectors S01, S02, S03, and S04, located at the bottom right (Figure 3). The PHT connector is for photo-resistive light sensors only (such as an LDR). The Borne-type connectors (with screws in green), located at the top and bottom left, are used to input and output power to the system. Table 2 explains the use of each connector.

The CPU (Central Processing Unit) used in the device is a Raspberry Pi 4. The CPU runs the Linux firmware described below. In addition, to reduce prototyping time with the Raspberry Pi, a “Pi hat” was attached to interface with various devices and submodules needed by the system. An RTC integrated circuit (IC) was used to provide time/date information. This IC will provide information via the I2C protocol, and its management will be performed automatically by the Linux-Raspberry driver. The purpose of the UPS is to provide power to the Raspberry Pi, its components, and the camera.

#### 2.1.2. Firmware

**Figure 4:**
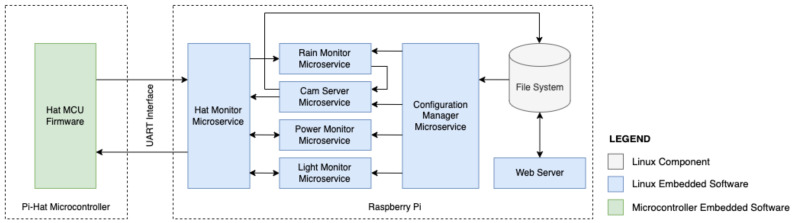
High-level firmware architecture.

A dual firmware approach was used, with a simple firmware for the microcontroller (MCU) (on Pi-Hat hardware) that performs non-preemptive tasks and handles analog and digital data polling and update requests, and a complex firmware for the microprocessor (MPU, on the Raspberry PI hardware) that runs an embedded Linux-based operating system (OS) that makes the requests to the MCU, processes its data, and sends back control values as a response. The high-level architecture of the technical solution is shown in Figure 4.

The MCU ports Sampled Values (SV), which are analog and digital signals from the environment (gathering from the Sensing Module), and act on Control Values (CV), which are digital signals that control device modules (such as LED status). Poll requests are sent from the MPU to the MCU, and all SVs are read instantly and sent back to the MPU in response. Then the MPU processes the values and responds to the MCU with CVs according to the current system configuration.

The set of embedded software running on the MPU side has different architectures. Unlike the MCU firmware, for each operational requirement, there will be a specific microservice responsible for generating an MC for a given SV.

A web server was developed to provide access to real-time visualization of the camera, to update settings, and to retrieve the video recordings, saved as MP4 files. Several open-source libraries were used and are presented here: nPair (used to communicate between the microprocessor firmware and the microcontroller firmware through the UART interface); ffmpeg (used to capture camera images); serde (used to serialize and deserialize system configurations); serde_json (used to serialize and deserialize system configurations); node libraries (such as express, dotenv, ejs, rstp-relay, and other dependencies were used to build the web server).

### 2.2. Capture Module

**Figure 5:**
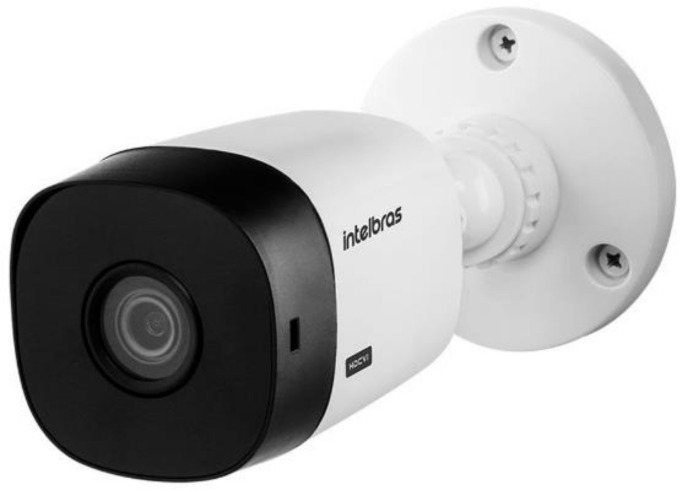
Camera used in the Capture module.

The capture module consists of a camera responsible for recording the erosion process during and immediately after a rainfall event. The camera used in the device is one of the IP (Internet Protocol) types, which means that the data related to the images is transported via the IP protocol. The device currently has an Intelbras G3 camera (Intelbras, São José, SC, Brazil) with an IP66 certification, which is resistant to strong jets of water and exposure to dust or other inclement weather (Figure 5). It should be noted, however, that any camera capable of using the RTSP streaming protocol can be used. It is strongly recommended that the camera be powered from an external source, as recommended by the manufacturer.

### 2.3. Sensing Module

**Figure 6:**
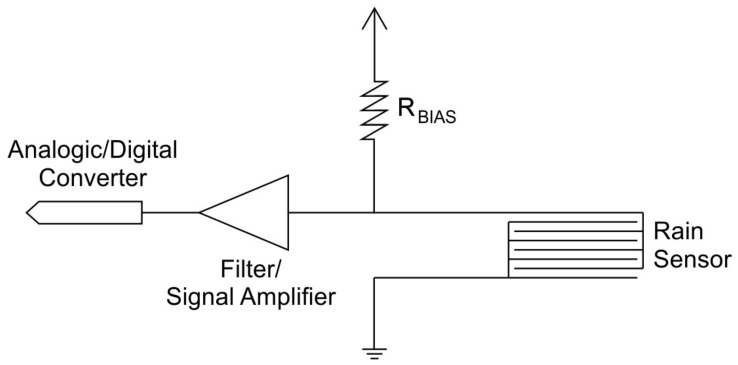
Electrical resistance measurement circuit for the rain sensor. Note the “voltage divider” configuration.

The rain sensors chosen for this project consist of a network of conductors lying on a plate made of insulating material. Under normal conditions, the electrical resistance of this system tends to be 10 MΩ. The rain monitoring system is based on measuring the electrical resistance across these conductors. When it rains, this resistance varies greatly and can reach values close to 1 kΩ, depending on the type of conductor used, the impurities diluted in the raindrops, and the volume of water poured over the sensor. In this way, a rain event can be detected, and the associated electrical signals can be processed. Figure 6 shows the idealized ohmic resistance measurement circuit for the sensor.

**Figure 7:**
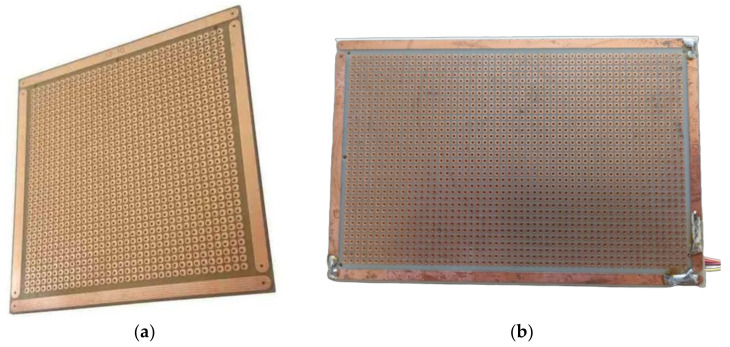
Rain sensor used in the sensing module: (**a**) FC-37 perforated universal plate; (**b**) mounted sensor, ready to use, with electrodes welded to the ends.

The rain sensor developed in this research consists of prototyping boards used for quick electronic component assembly (Figure 7). These boards have copper traces on the insulating material (usually fiberglass). The average distance between the tracks and the holes is 2.54 mm. When the environment is dry, the sensor output is in a high-impedance state, and the analog-to-digital converter reads a maximum voltage value (relative to the system supply voltage). If there is moisture between the sensor grids, the bias resistor forms a voltage divider with the sensor, which now has a relatively low ohmic resistance. This voltage change is detected and indicates the possibility of a rain event. Figure 7 shows the rain sensor model developed for this study in terms of rain event detection (Figure 6). The sensor has an operating voltage of 3.3 V to 5 V and a maximum current of 100 mA. The rain sensor has dimensions of 15 × 10 cm, is 1 mm thick, the control boards are 2.1 × 1.4 cm, and the cable is between 20.0 cm and 2.0 m long.

For rain sensors, the following considerations must be made to estimate the values of each field: Rain detection sensors are of the resistive type, i.e., they range from a typical resistance of 10 MΩ to 1 kΩ (or less). The detection circuit has a characteristic output impedance (R_BIAS_, shown in Figure 6) of 10 kΩ (ten kilo ohms). The analog-to-digital converter is capable of reading values from 0 to 1023, where 0 represents an impedance equal to or greater than 3 MΩ and 1023 represents the minimum impedance (or a short between the sensor terminals). The gain value is multiplied by the value read from the ADC. For example, if the gain is 2.5, this means that the value considered for the enable or disable logic is from 0 to 2557.5 (and not from 0 to 1023). It is recommended to keep the gain at unity (with a value of 1). Upper and lower limits are used for system hysteresis. This prevents oscillations and false detections. The sensor is considered wet (i.e., it has detected raindrops) only if the sampled values are above the upper limit, and the sensor is considered dry if they are below the lower limit. If the sensor is faulty or has been removed, it is recommended to disable that sensor in device settings.

**Figure 8:**
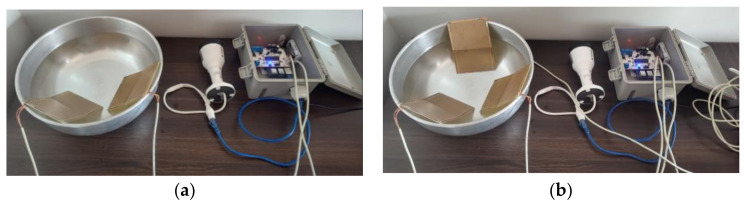
Execution and validation of tests to determine the configuration impedance of rain sensors for different wetting and drying scenarios: (**a**) two rain sensors; (**b**) three rain sensors.

To determine the upper and lower limits, use a multimeter to measure the electrical resistance between the sensor terminals when the sensor is dry or slightly moist; when the sensor is submerged in water or very moist, use the same multimeter to measure the resistance. The test can be repeated several times to obtain a significant number of samples (Figure 8). 

Consider using the average or highest value of each sample. It is also worth noting that there must be at least 100 kΩ between samples for the hysteresis of the system to work as desired. Equation (1) should then be used to estimate the upper limit (wet sensor) and lower limit (dry sensor) values:

(1) $$\mathrm{Limit}=\frac{1023\cdot R}{10,000+R}$$

where *R* is the resistance in kΩ.

The light sensor schematic follows the same pattern and calculation formula as the rain sensors in the previous section. Since the use of this sensor is reserved for future use, it is recommended that this sensor be disabled.

## 3. Results and Discussion

### 3.1. Hardware

**Figure 9:**
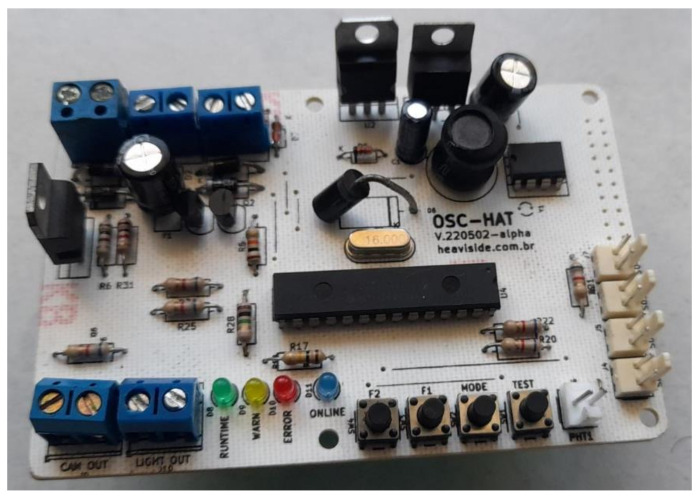
Printed board (Pi-Hat).

The final version of the developed Raspberry-type Pi-Hat hardware is shown in Figure 9. It shows the Pi-Hat board with connectors for the sensor interface and the LEDs to inform the device status. A set of PTH (pin-through hole) and SMT (surface mount technology, assembled on the bottom of the board) were used due to board space constraints. The main microcontroller is a Microchip ATMega328P. These ADC (Analog to Digital Converter) input pins are attached to all sensor outputs (including rain sensors, light sensors, and battery voltage sensors). The other digital pins are attached as outputs to LEDs and as inputs to tactile switch buttons. A power regulation IC provides a conversion of 13 V from the battery or 15 V from the external power adaptor to the 5 V required by the Raspberry. The RTC IC has a battery (on the bottom layer of the board, not shown in Figure 9) to keep time and date even if the system is powered off. The borne-type connectors named “CAM OUT” and “LIGHT OUT” are used to provide power to the camera of the capture module and to a light source (to provide nightly video recordings). The connectors on the top-left corner are for power input from a power adaptor, from a battery, and from a photovoltaic panel.

### 3.2. Firmware (Embedded Software)

**Figure 10:**
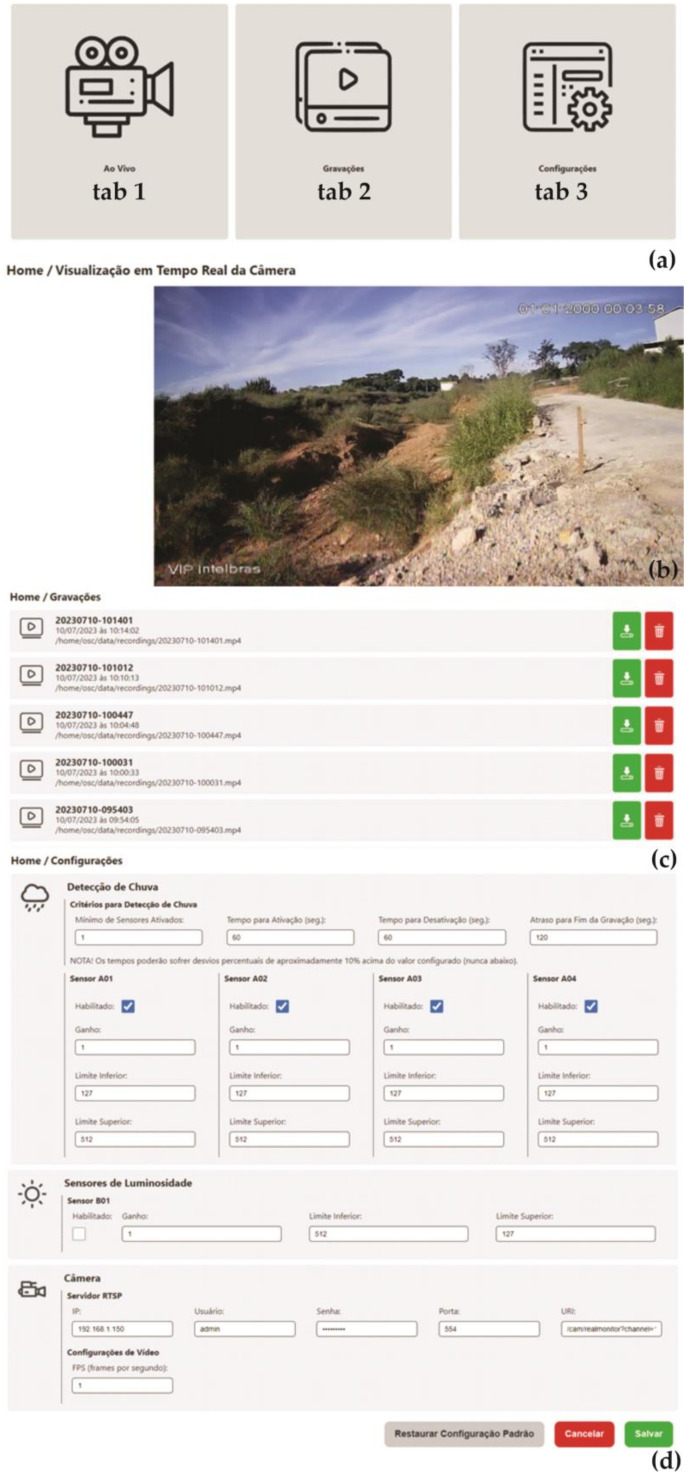
Main software screens: (**a**) Home screen; (**b**) Real-time visualization; (**c**) Stored recordings; (**d**) Settings. All texts on the screens presented are in Portuguese, the official language in which the firmware was developed.

Figure 10 shows the main screen of the software and its three tabs. The first tab takes you to the camera’s snapshot (Figure 10b). The second tab takes you to the previous recordings stored on the device (Figure 10c). The third tab is the sensor and recording settings screen (Figure 10d).

The first tab (Figure 10b) is for viewing the images captured by the camera in real-time. It is important to note that due to the processing limitations of the minicomputer used in the system, the images will show a delay or lag, and may also be blurred as the camera moves and adjusts. However, when the camera stabilizes, the image is displayed correctly. Although the images may be of poor quality, the images recorded in archives (i.e., those saved in a file) are of the highest quality of the camera (usually HD quality). Low quality only occurs during “live” broadcasts.

The Recordings tab is used to view the video files recorded by the system during rain events (Figure 10c). Each card displayed is a video recorded in the system’s memory. The video cards display the title of the video (in TIME-DATE format), the day and time it was recorded, and finally the folder path to the file.

**Figure 11:**
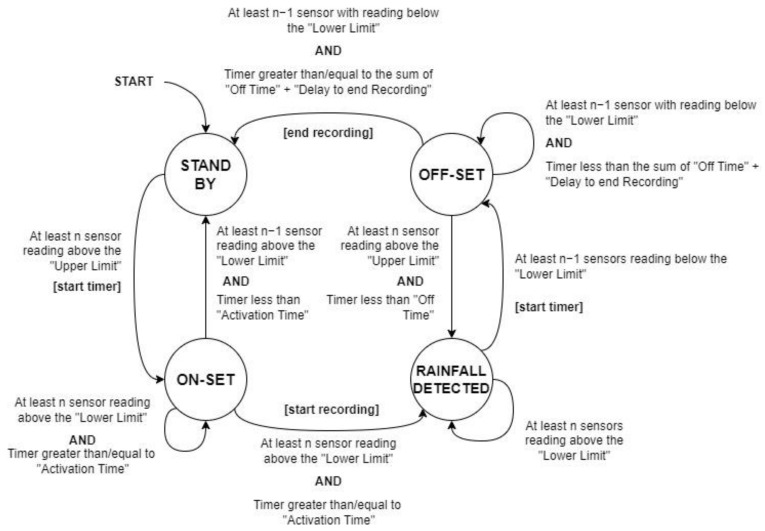
State Machine Diagram.

In the Settings tab, you can configure the activation, deactivation, and post-recording delay times, called Rain Detection Criteria, for the sensors and the camera. Figure 11 illustrates the understanding of each of the times on the Rain Detection Criteria Settings screen. In addition to the times, it is possible to configure the sensitivity of the rain sensors, adjusting it as presented in the methodology. As soon as the number of sensors in the “Minimum Activated Sensors” field shows a signal indicating rain, the system enters the rain pre-detection mode. After the “Activation Time” has elapsed (in minutes), if the number of sensors continues to detect rain, the system switches to rain detection mode and starts recording. If the number of activated sensors is less than the “Minimum Activated Sensors”, the system will enter post-detection mode. To confirm that the rain has stopped, the number of active sensors must remain below the “Minimum number of active sensors” for the time specified in “Deactivation time”. Once the end of the rain is confirmed, the “End Recording Delay” time will keep the camera on for that period.

Currently, the only information related to the camera used in the erosion monitoring equipment that can be changed is that related to the RTSP server, and it depends on the model of the camera used as well as the information available in the manufacturer’s manual for this component, so it is recommended not to change this parameter.

### 3.3. Validation of Monitoring Times

**Figure 12:**
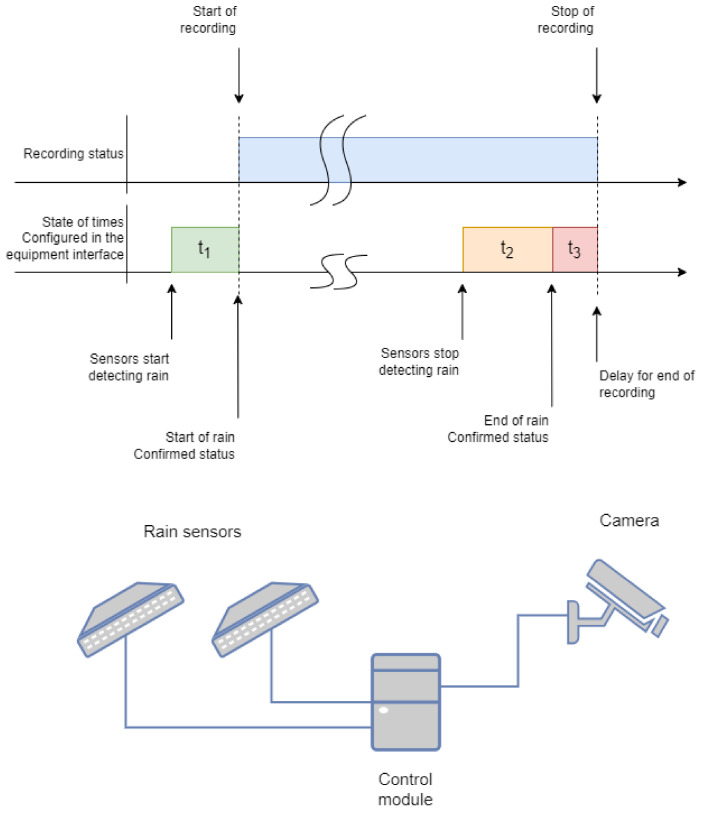
Time configuration for the capture, sensing, and control modules.

Figure 12 provides an understanding of the response times that the equipment should have and helps you understand how the configured times work and how they relate to the events detected by the rain sensors.

Table 3 shows the results obtained to evaluate the response times of the equipment in terms of activation, deactivation, and delay at the end of the recording.

Table 3 shows good agreement between the theoretical and experimental activation and deactivation times. Time 1 (*t*_1_) refers to activation, and *t*_2_ refers to deactivation. This agreement is consistent across all three sensors (Sensors 1, 2, and 3). Overall, the coefficient of variation remains low when comparing the times of the same values. The coefficient of variation only increases in cases where there is a progression in the reference value. This allows us to conclude that the activation, deactivation, and delay times are in line with what was proposed, showing that the programming performed in the hardware is working well.

It is noteworthy that the low post-rain time values used (Table 3) were only used in the calibration and validation process of the rain sensors to check whether they sent correctly, quickly, and efficiently the rain detection termination signal.

However, it is suggested that this post-rain time be defined, is the responsibility of the user, and can be adjusted according to the specific characteristics of each type of erosion to be monitored and the rainfall regime of the region.

Table 4 suggests a decrease in resistance with more connections of the sensors in each gate. This phenomenon is likely due to the parallel nature of the circuit. When more sensors are connected, the total resistance decreases because the current has multiple pathways to flow through. The standard deviation of resistance across individual gates was 1292.1 and 2039.1 when the sensor was partially wet, indicating significant variation.

However, when the rain sensor was completely wet, the standard deviation dropped to 517.7 and 65.4, suggesting increased consistency and reliability in the measurements. This finding is significant because it shows how important it is to calibrate the rain sensors developed for all possible wetting and drying ranges, the influence of their installation inclination, and the evaluation of whether or not noise signals appear during the operation phase at high ambient temperatures, that is, around 50 °C. All these phases of field tests will be carried out, and their results and validation will be presented in future work, along with the discussion on the quality and geotechnical interpretation of the videos and images captured by the OSC equipment during rainy times.

### 3.4. Erosion Monitoring Equipment (OSC)—Complete

**Figure 13:**
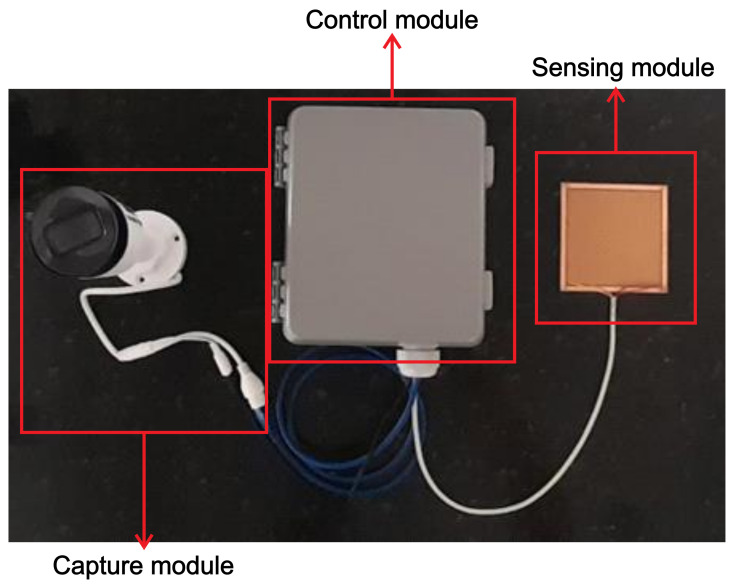
Products: capture, sensing, and control modules.

After all the methodological steps of idealization, planning, assembly, operation test simulation, and validation of the system and sensors, it was possible to allow the use of the erosion monitoring equipment in the field, called OSC. Figure 13 shows the product of the three modules that constitute it, namely: control, sensing, and capture.

However, before using the OSC equipment in the field, we tested it in conjunction with an experimental rainfall simulator (RS) developed by Mendes [20] and Mendes et al. [8]. This RS is capable of creating several artificial rain scenarios to evaluate various parameters of the OSC’s functionality, operation, and weather resistance. The test summations in the RS also made it possible to evaluate the quality of the videos and images generated by the OSC, as well as to test, measure, and validate the activation and deactivation programming times of the camera and rain sensors.

**Figure 14:**
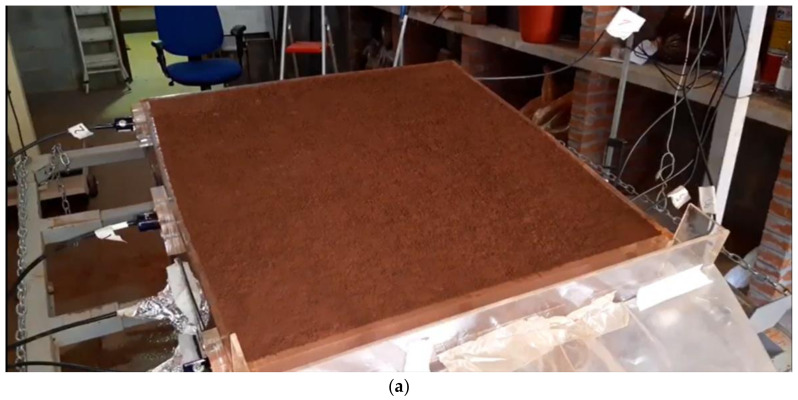

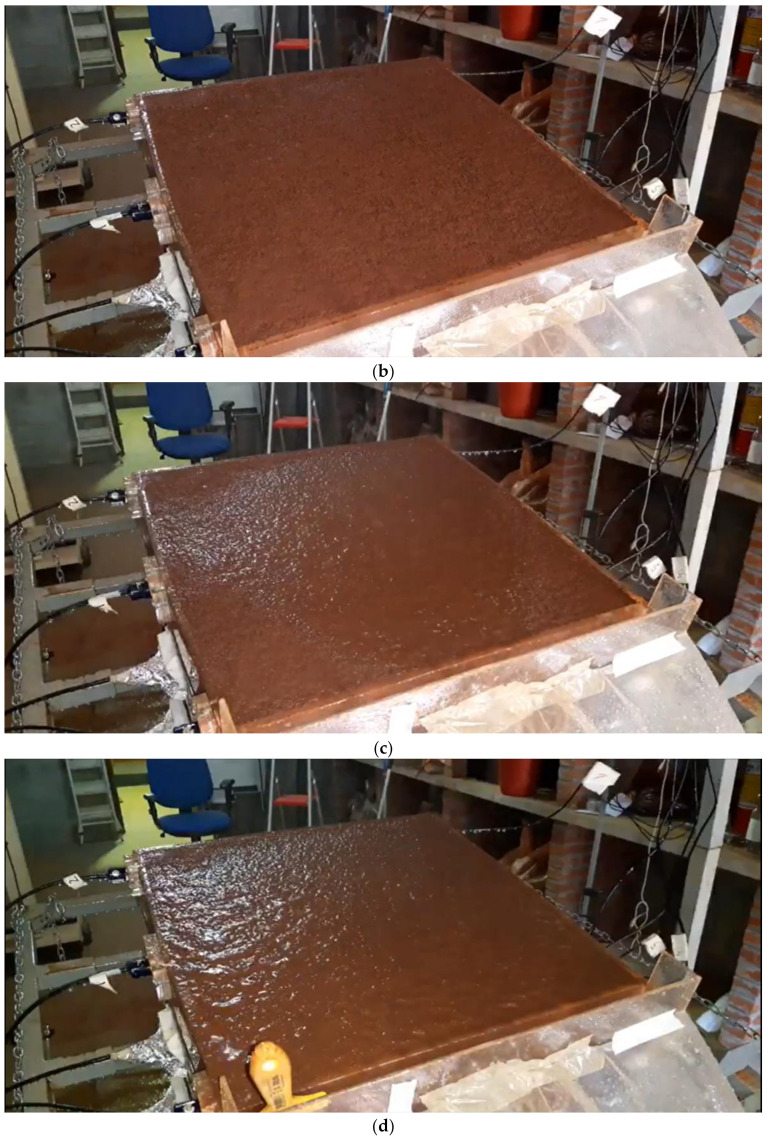
Steps and details of monitoring the test carried out in RS for exposed soil (sample surface). Test considering simulated rainfall of 223.0 mm h^−1^, initial soil moisture of 25%, and α = 15°: (**a**) overview of the RS before the test; (**b**) 38 s; (**c**) 76 s; (**d**) 152 s after the start of the test.

In Figure 14, it is possible to evaluate the results of the images generated by the OSC during one of the rainfall events generated by the experimental rainfall simulator (RS) developed by Mendes [20] and Mendes et al. [8].

Evaluating Figure 14, it is clear that the quality of the images recorded by the OSC is satisfactory to evaluate how the soil detachment and transport process begins in the sample plot, that is, how the beginning of the erosion process is triggered.

Through the videos and images captured by the OSC equipment (Figure 14), the influence of rainfall intensity on the formation of erosion processes is evident (Figure 14b). Furthermore, the impact of raindrops can be observed when they hit the ground, as well as the phenomenon known as the splash effect (Figure 14c). The initiation and propagation of surface flows (runoff), both low and high-speed, along the inclined surface of the soil is also noticeable. This analysis allows us to clearly identify the formation and evolution of the preferential paths of erosion features (Figure 14d), the main objective of OSC.

**Figure 15:**
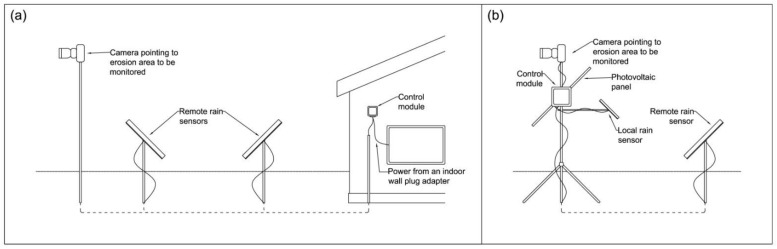
Versatility of installing and powering the OSC in the field. (**a**) through electrical energy; (**b**) through solar energy.

The installation of the three equipment modules in the field can be adjusted for different scenarios, allowing them to be integrated together or apart as necessary. It is important to adapt to the various difficulties that may arise in the field, such as large distances between the electrical supply point and the erosion site, places that are difficult to access or with unsuitable soil for fixing the sensor rods and support, and large distances between erosion and rain sensors, among other challenges (Figure 15).

In general, the configuration and installation of equipment for monitoring erosion in the field do not present additional concerns. The equipment can be powered by the conventional electrical network (100 to 220 VAC) or by a photovoltaic panel (Figure 15), and programming the rain monitoring times is simple and intuitive through the developed firmware, that is, by the module control (Figure 10).

**Figure 16:**
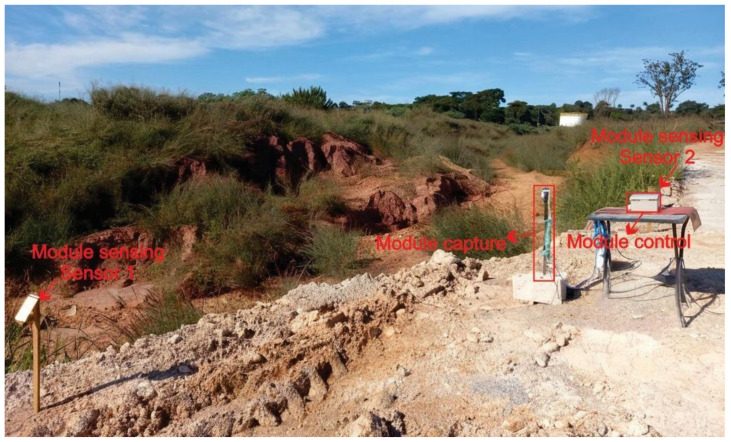
Overview of erosion: study area used to install the OSC, located in the city of Goiânia, state of Goiás (16°42′30.1″ S 49°11′22.1″ W), Brazil, on 20 April 2024.

Regarding the installation of the equipment in the field, both the issue of wind and the location of the three existing modules were considered, proposing adjustable fixing systems depending on the actual field condition that can be fixed or supported on any type of structure, such as a concrete or masonry wall, a metal or wooden table, or metal rods (Figure 15 and Figure 16). The only limitation is the length of the electrical and data cables, which is up to 30 m. All components are water-protected by IP65-rated devices, eliminating the need for additional protection.

Therefore, monitoring erosion processes in the field is an important task, especially in the implementation and operation phase of large projects, such as urban subdivisions, that is, the type of study area used for future analysis of videos about the erosion process. formation and development of erosion resulting from rainwater releases (Figure 16).

In relation to the information obtained, that is, the images of erosion and the formation process during rainfall events captured by the proposed monitoring equipment, it is highlighted that the objective of this study is not to quantify the volume of eroded soil or the area affected by the erosion. The focus is to understand how the preferred paths and routes of rainwater are initiated, processed, and developed for each type of soil, slope, vegetation cover, and rainfall intensity involved in each monitored erosion.

It is important to highlight that the rain sensors developed for this equipment do not measure the rainfall intensity of events, as there are numerous types, brands, and manufacturers of these sensors available. The function of the sensors in this equipment is only to detect the occurrence of rain, activate the equipment, and start recording. The relationship and magnitude of rainfall intensity must be obtained through the closest official meteorological stations or satellite data.

The use of OSC will allow us to observe relevant details, whether atmospheric, hydrological, or geotechnical, focusing mainly on how surface runoff is formed and propagated, influenced by the slope and type of soil, to develop preferential paths for the formation of erosive features. This type of monitoring is essential to understanding the dynamics of erosion processes and supporting prevention and mitigation actions.

## 4. Conclusions

The use of monitoring equipment for hydraulic and geotechnical works is common in engineering. However, monitoring erosion processes, whether initial or developing, especially with regard to river erosion or those caused by deficiencies in rainwater drainage, is not common. 

Thus, the main objective of this study was to idealize, plan, develop, and validate equipment to monitor the process of erosion development in the field when rainfall events of different intensities occur, called OSC equipment.

Considering that erosion processes depend on different variables and parameters involved, be they physical, hydrological, or geotechnical, the aim of developing and using the OSC is to be able to correlate them in the future in order to improve the physical understanding of the phenomenon. 

According to the results obtained, all phases of OSC development were developed, presenting the characteristics and functioning of the materials, microcontrollers, regulation and power supply systems, and electronic hardware circuits, as well as the performance and validation of the firmware and the sensors involved, presenting satisfactory operation depending on the values on the metrics presented, and can therefore be used in different climatic conditions in hydraulic and geotechnical works.

Based on the results, the footage obtained from experimental laboratory samples during simulated rainfall events proved to be satisfactory considering aspects of programmable times and image quality during the day, without the need for artificial lighting to analyze the development of erosive processes. 

Additionally, this study has potential limitations. The study and results focus mainly on the planning and development of the equipment itself and not on the quality and analysis of images and videos generated of erosion processes during and after rain events, which are suggestions for future work. 

Short- and long-term observations and field investigations (geotechnical characterization, such as granulometry and soil erodibility), influenced by the existing slope, can also help to obtain a better understanding of the development of erosion processes, soil disintegration, and deposition under different environmental conditions. In practice, the equipment for monitoring erosive processes in the field (OSC) will be the means by which a database of images and videos can be acquired, fed, and updated—that is, a dataset that can be used as input data for work with different artificial intelligence (AI) tools in the area of computer vision and data science. In the future, it is expected to consider rainfall events that occur at night.

## Figures and Tables

**Table 1:** Status of each LED in use.

LED	Description	Indication
Green	RUNTIME	When it flashes twice every 5 s, it indicates that the system is operational and scanning the sensors.
Yellow	WARN	If accessed, this indicates a low battery in the system.
Red	ERROR	If accessed, this indicates that the camera is not connected.
Blue	ONLINE	When accessed, this indicates that the system is recording video.

**Table 2:** Description of each terminal block connector.

Borne	Description	Use
J1	POWER IN	Power input. Use a 15 V-1 A Direct Current power supply.
J2	AUX	Auxiliary power input. Suitable for use with
J2	POWER IN	Solar panel 24 V-500 mA.
J3	BATTERY	12 V 1300 mA lead-acid battery connection.
J9	CAM OUT	Auxiliary power output for the camera
J10	LIGHT OUT	Auxiliary power output for night lighting. Reserved for future use. Do not connect anything to these terminals.

**Table 3:** Rain sensor response times.

Number of Sensors	Activation (s)	Deactivation (s)	Delay—End of Recording (s)	*t*_1_ (s)	*t*_2_ (s)	*t*_3_ (s)
1	30	60	120	59	100	77
	60	60	120	81	102	84
	120	60	120	131	91	86
	180	60	120	210	131	115
			Mean	120	106	91
			Std. Dev.	58	15	15
			COV	48%	14%	16%
	60	30	120	87	71	56
	60	60	120	81	102	84
	60	120	120	89	161	146
	60	180	120	97	221	218
			Mean	89	139	126
			Std. Dev.	6	57	62
			COV	6%	41%	49%
	60	60	30	95	101	81
	60	60	60	79	101	95
	60	60	120	81	102	84
	60	60	180	99	101	93
			Mean	89	101	88
			Std. Dev.	9	0	6
			COV	10%	0%	7%
3	60	60	120	83	115	101
2	60	60	120	91	107	98
1	60	60	120	81	102	84
			Mean	85	108	94
			Std. Dev.	4	5	7
			COV	5%	5%	8%
3	120	60	120	148	127	119
2	120	60	120	135	102	90
1	120	60	120	131	91	86
			Mean	138	107	98
			Std. Dev.	7	15	15
			COV	5%	14%	15%
3	60	120	120	91	180	145
2	60	120	120	80	170	160
1	60	120	120	89	161	146
			Mean	87	170	150
			Std. Dev.	5	8	7
			COV	6%	5%	5%
3	60	60	60	90	120	110
2	60	60	60	91	111	93
1	60	60	60	79	101	95
			Mean	87	111	99
			Std. Dev.	5	8	8
			COV	6%	7%	8%

where: *t*_1_ is the time to start recording (i.e., the time for the blue light to turn on), *t*_2_ is the time of detection of the end of rain by the sensors, *t*_3_ is the time to stop recording (i.e., the time for the blue light to turn off), Std. Dev. is Standard Deviation, and COV is coefficient of variation.

**Table 4:** Resistance values, R (Ω) for dry, partially wet, and completely wet conditions.

Number of Sensors		Dry	1/2 Wet	Wet
1	Gate 1	0.0	12,834.8	19,652.2
2	Gate 1	0.0	11,446.5	18,027.4
	Gate 2	0.0	7368.4	16,992.1
	Mean	0.0	9407.5	17,509.7
	Std. Dev.	0.0	2039.1	517.7
	COV	-	22%	3%
3	Gate 1	0.0	10,257.4	13,197.3
	Gate 2	0.0	7107.0	13,250.0
	Gate 3	0.0	8944.4	13,092.6
	Mean	0.0	8769.6	13,179.9
	Std. Dev.	0.0	1292.1	65.4
	COV	-	15%	0%
Mean		0.0	9659.8	15,701.9
Std. Dev.		0.0	2078.0	2638.5
COV		-	22%	17%

## Data Availability

Data are contained within the article.

## Abbreviations

Universal Soil Loss by Erosion (USLE), Human Machine Interface (HMI), Single Board Computer (SBC), Uninterruptible Power Supply (UPS), Real Time Clock (RTC), microcontroller (MCU, on Pi-Hat hardware), Central Processing Unit (CPU), integrated circuit (IC), microprocessor (MPU, on the Raspberry PI hardware), operating system (OS), Sampled Values (SVs), Control Values (CV), IP (Internet Protocol), Pin-Through Hole (PTH), Surface Mount Technology (SMT), ADC (Analog to Digital Converter), ADC (Analog to Digital Converter) and, Erosion monitoring equipment (OSC).
